# Serum Antioxidant Vitamins Mediate the Association between Periodontitis and Metabolically Unhealthy Overweight/Obesity

**DOI:** 10.3390/nu14224939

**Published:** 2022-11-21

**Authors:** An Li, Zhi Tang, Peijun Zhu, Florien van den Bosch, Yuntao Chen, Shulan Xu, Geerten-Has E. Tjakkes

**Affiliations:** 1 Center of Oral Implantology, Stomatological Hospital, Southern Medical University, Guangzhou 510280, China; 2 Department of Periodontology, Center for Dentistry and Oral Hygiene, University Medical Center Groningen (UMCG), University of Groningen, 9713 AV Groningen, The Netherlands; 3 Department of Stomatology, Zhujiang Hospital, Southern Medical University, Guangzhou 510280, China; 4 Department of Epidemiology and Public Health, University College London, London WC1E 6BT, UK; 5 Medical Statistics and Decision-Making, Department of Epidemiology, University Medical Center Groningen, University of Groningen, 9713 AV Groningen, The Netherlands

**Keywords:** metabolic syndrome, overweight and obesity, periodontitis, serum antioxidants, systemic inflammation

## Abstract

Background: Periodontal disease is associated with metabolic syndrome and obesity. This cross-sectional study aimed to investigate whether serum antioxidant vitamins could mediate the association between periodontitis and a metabolically unhealthy phenotype in the overweight and obese population; Methods: We included 6158 Americans (body mass index (BMI) ≥ 25 kg/m^2^) from the Third National Health and Nutrition Examination Survey (NHANES III). Periodontitis was defined using a half-reduced CDC/AAP (Centers for Disease Control and Prevention/American Academy of Periodontology) definition. Having two or more metabolic abnormalities was defined as a metabolically unhealthy overweight and obese (MUO) phenotype. Mediation analysis of four oxidative stress biomarkers (serum antioxidant vitamins A, C, D, and E) was conducted; Results: Of participants with overweight and obesity, 2052 (33.3%) Americans were categorized as having periodontitis. Periodontitis increased dyslipidemia risk and systemic inflammation in the overweight and obese population. In the multivariable logistic regression model, periodontitis was positively associated with MUO (adjusted odds ratio = 1.238; 95% confidence interval: 1.091 to 1.406). These findings were validated in an independent cohort. Serum vitamins C and D were estimated to mediate 19.3% and 8.4% of the periodontitis–MUO association. Conclusions: Periodontitis might decrease serum vitamins C and D and induce a metabolically unhealthy state among adults with overweight and obesity.

## 1. Introduction

Overweight or obesity has become a global health problem due to its increasing prevalence and disease burden in recent decades [1]. The national prevalence of overweight (body mass index (BMI) ≥ 25 kg/m^2^) and obesity (BMI ≥ 30 kg/m^2^) in the United States (US) population will be approximately 80% and 50%, respectively, by 2030 [2]. In the individuals with overweight and obesity, overburdened adipose tissues initiate complex interactions between genetic, environmental, and neuroendocrine factors, leading to chronic low-grade inflammation and systemic metabolic dysfunction [3]. Obesity has been suggested to play a critical role in increasing cardiometabolic morbidity and mortality [4]. However, obesity may not always mean being unhealthy. Approximately 30% of people with obesity in the US exhibited normal insulin sensitivity and favorable metabolic profiles [5], categorized as the metabolically healthy overweight and obese (MHO) phenotype [6]. Compared to those with the metabolically unhealthy overweight and obese (MUO) phenotype, individuals with MHO are associated with a lower risk of cardiometabolic disease and mortality [7,8,9].

Periodontitis is a common inflammatory disorder in the oral cavity that can induce metabolic alterations via host immune responses and systemic inflammation [10]. Pathological activities caused by periodontitis may lead to metabolic–inflammatory conditions, such as obesity and related comorbidities (diabetes and metabolic syndrome (MetS)). These comorbid conditions might even partly explain the effect of periodontitis on mortality [11]. Thus, the intertwined relationships between periodontitis, obesity, and metabolic disorders have attracted significant attention over the years [12]. A recent cross-sectional study showed that severe periodontitis was associated with MetS in the general population in Spain [13]. Compared to non-obese controls with metabolic health, MUO was significantly associated with severe periodontitis [13].

Vitamin dysregulation, especially of B-group vitamins and of C and D vitamins, might play an indispensable role in periodontal health via increased oxidative stress [14]. In turn, periodontal inflammation has been reported to induce oxidative stress in gingival crevicular fluid and saliva as well as attenuate local and systemic total antioxidant capacity [15]. Low-grade inflammatory status related to periodontal pathogens could initiate or aggravate inflammatory comorbidities by enhancing oxidative stress [16,17]; specifically, periodontitis-associated oxidative stress and mitochondrial dysfunction have been regarded as potential contributors to the pathophysiology of cognitive impairment [18], renal dysfunction [19], and abnormalities of glycemic status [20]. More importantly, vitamin D deficiency could affect antioxidant defense and oxidative stress balance in obesity-related metabolic disorders, through disturbing adipocytokines secretion, metabolism, and lipid storage [21]. However, the oxidative stress mechanisms linking periodontitis and MUO have not been well studied. This study aimed (1) to determine the association between periodontitis and a metabolically unhealthy phenotype in the population with overweight and obesity; (2) to explore the mediating effect of serum antioxidant vitamins on the association.

## 2. Materials and Methods

### 2.1. Study Population

Data on participants in the Third National Health and Nutrition Examination Survey (NHANES III, 1988–1994) were investigated in this cross-sectional study. We regarded NHANES III as the discovery dataset. In addition, the Korea National Health and Nutrition Examination Survey (KNHANES) 2015 was regarded as the validation dataset. These two population-based surveys were serial cross-sectional surveys using a complex, multistage probability design [22,23]. Data related to health and nutritional status were collected from household interviews, mobile physical examination, and laboratory tests. All NHANES and KNAHENS participants provided written informed consent in the original data collection. The present study using de-identified data with no direct participant contact was not considered a human subject’s research. Thus, this analysis was exempt from an institutional review board ethics review according to the National Institutes of Health policy.

**Figure 1:**
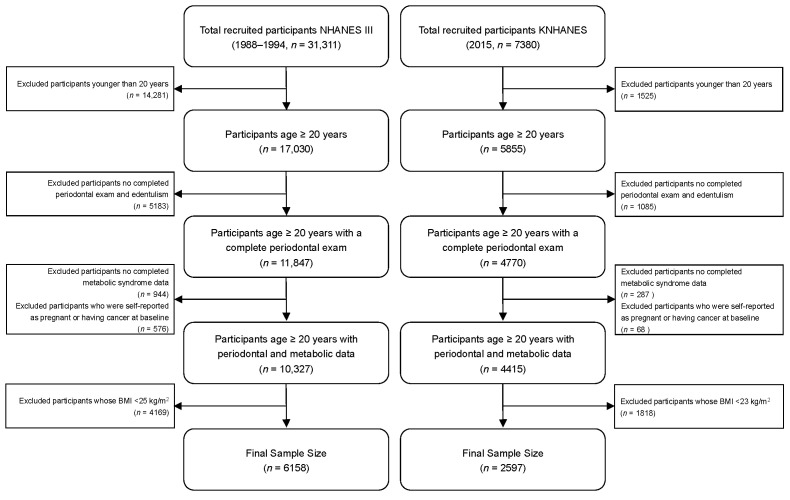
Flow chart of final sample from The Third National Health and Nutrition Examination Survey (NHANES III) and Korean National Health and Nutrition Examination Survey (KNHANES).

The exclusion criteria were (1) age < 20 years (NHANES, *n* = 14,281; KNHANES, *n* = 1525); (2) participants without complete periodontal data or edentulous participants (NHANES, *n* = 5183; KNHANES, *n* = 1085); (3) participants without complete metabolic syndrome data (NHANES, *n* = 944; KNHANES, *n* = 287); (4) participants with cancer or pregnant participants (NHANES, *n* = 576; KNHANES, *n* = 68); (5) BMI < 25 kg/m^2^ in NHANES III (*n* = 4169) and BMI < 23 kg/m^2^ in KNHANES 2015 (*n* = 1818). Finally, this analysis included 6158 participants with overweight and obesity in NHANES III (discovery dataset) and 2597 in KNHANES 2015 (validation dataset), see Figure 1.

### 2.2. Periodontal Clinical Parameters

In the discovery dataset (NHANES III), trained and calibrated examiners selected two random quadrants (one in the maxilla and one in the mandible) to determine the periodontal parameters based on a partial-mouth periodontal examination protocol. Attachment loss (AL), probing pocket depth (PPD), and bleeding on probing (BOP) were assessed at mid-buccal and mesio-buccal sites per tooth [24]. As the periodontal examination excluded the extracted third molars, a maximum of 14 teeth and 28 sites per participant could be examined. Dentition examination recorded the number of missing teeth. Periodontal status was assessed using the half-reduced case definition of CDC/AAP (Centers for Disease Control & Prevention and American Academy of Periodontology) [25,26]. Moderate/severe periodontitis was defined as ≥ 1 inter-proximal site with ≥ 4 mm AL or ≥ 1 inter-proximal site with ≥ 5 mm PPD; the others were defined as no or mild periodontitis and set as a reference.

In the validation dataset (KNHANES 2015), the World Health Organization (WHO) community periodontal index (CPI) was used to assess periodontal status. The dentition was divided into sextants. The index teeth were used for the examination: #11, #16, #17, #26, #27, #31, #36, #37, #46, and #47. The CPI was scored from 0 to 4 as follows: 0 (healthy), 1 (gingival bleeding), 2 (calculus), 3 (PPD: 3.5–5.5 mm), and 4 (PPD > 5.5 mm). In this study, CPI scores of 0, 1, and 2 were defined as non-periodontitis, whereas scores ≥ 3 in any sextant indicated periodontitis, as described previously [27].

### 2.3. Metabolic Health Assessment

MUO was defined as a combination of BMI and metabolic health. Metabolic health parameters include blood pressure (BP), fasting blood glucose, triglycerides, high-density lipoprotein (HDL) cholesterol, homeostasis model assessment of insulin resistance (HOMA-IR), and C-reactive protein (CRP). Systolic and diastolic BP was measured according to a standardized protocol, and the mean of three consecutive readings was used in this analysis. Serum fasting glucose, triglyceride, HDL cholesterol, fasting insulin, and CRP concentrations were measured using standardized laboratory procedures. HOMA-IR was calculated using fasting blood glucose and insulin:

(1) HOMA−IR =fasting glucose mg/dL×fasting insulin IU/mL450

Overweight and obesity for the American and Korean populations was defined as a BMI of ≥25 kg/m^2^ and ≥23 kg/m^2^, respectively [28]. Participants with maximally one of the following metabolic abnormalities were defined as the MHO phenotype, whereas those with ≥2 components were defined as the MUO phenotype [29,30]. The metabolic abnormalities included (1) hypertension: systolic BP > 130 mmHg or diastolic BP > 85 mmHg or antihypertensive medication use; (2) hyperglycemia: fasting blood glucose ≥ 100 mg/dL or antidiabetic medication use; (3) elevated triglycerides: triglycerides ≥ 150 mg/L or anti-cholesteremic medication use; (4) reduced HDL cholesterol: HDL cholesterol < 40 mg/dL for males or < 50 mg/dL for females or anti-cholesteremic medication or agent use; (5) insulin resistance: homeostasis model assessment of insulin resistance (HOMA-IR) ≥ the 90th percentile; (6) systemic inflammation: CRP ≥ the 90th percentile.

### 2.4. Serum Antioxidant Vitamins

The present study regarded the serum antioxidant vitamins as the biomarker of oxidative stress. The serum antioxidants assessed were vitamins A, C, D, and E in the NHANES III dataset. Serum vitamins A, C, and E concentrations were determined by isocratic high-performance liquid chromatography; the DiaSorin assay measured serum vitamin D levels. Detailed laboratory procedures are provided elsewhere (https://www.cdc.gov/nchs/data/nhanes/nhanes3/cdrom/nchs/manuals/labman.pdf; accessed on 30 September 2022). Serum vitamin A (μg/dL), vitamin C (mg/dL), vitamin D (nmol/L), and vitamin E (μg/dL) were treated as continuous variables and transformed as Z-scores in the mediation analyses.

### 2.5. Covariates

The following covariates were selected because they were hypothesized to be related to periodontitis, metabolic health, and obesity [13,31]. Covariates consisted of socio-demographic variables, lifestyle behaviors, self-reported chronic diseases, and laboratory sample data. Sociodemographic variables included age (continuous) and gender (male and female). Only in the NHANES III, the race/ethnicity was recorded as non-Hispanic white, non-Hispanic black, Mexican American, and others. Family income (high, middle, or low) and educational attainment (≤ primary school, middle or high school, or ≥ college) were regarded as indicators of socioeconomic status. Smoking status (never, former, and current) and diagnosed diabetes, hypertension, and cardiovascular diseases were also obtained from the interviews. Serum biochemical parameters included glycohemoglobin A1c, total cholesterol, and creatinine.

### 2.6. Statistical Analyses

The present study examined the association between periodontitis and MUO in the discovery dataset (NHANES III) and the validation dataset (KNHANES 2015). To accommodate the complex design of the NHANES survey, we incorporated sample weights, clustering, and stratification to generate nationally representative population estimates. We calculated counts (percentage (%)) of categoric variables, means (standard deviation (SD)) of parametric continuous variables, and median (interquartile range (IQR)) of non-parametric continuous variables. Independent t-test and chi-square tests were used for the comparisons of continuous and categorical variables, respectively. Survey-weighted multivariable logistic regression models were developed to investigate the associations of periodontitis (exposure) with the metabolically unhealthy phenotype and its individual components (outcome) via odds ratios (OR) and corresponding 95% confidence intervals (CI). The univariable model was not adjusted for confounders. Multivariable models were adjusted for age, gender, race/ethnicity (only NHANES III), educational level, income level, smoking status, total cholesterol, creatinine, the missing teeth, and heart disease. The effect modification was tested for age, gender, or smoking status by including a multiplicative interaction term in the model.

Several sensitivity analyses were performed to test the robustness of the results. First, the metabolic phenotype was assessed by the International Diabetes Federation (IDF) consensus definition [32]. The MUO was defined by ≥2 cardiometabolic disorders (including hypertension, high fasting plasma glucose, elevated triglyceride levels, and reduced HDL cholesterol) among adults with overweight and obesity. Others were defined as MHO [13]. Second, individuals with diabetes or cardiovascular disease were excluded because they could affect periodontitis and the metabolic phenotype. Third, the population with obesity (BMI ≥ 30 kg/m^2^ in the US or BMI ≥ 27.5 kg/m^2^ in Korea) was selected to assess the relationship between periodontal health and the metabolic phenotype. Fourth, we furtherly adjusted the number of vitamins and minerals taken in the NHANES III. Fifth, the current smokers (*n* = 1431) were excluded.

**Figure 2:**
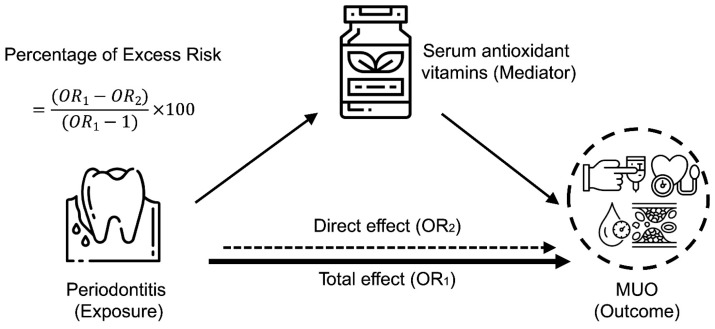
Mediating effect of oxidative stress on the relationship between periodontitis and metabolically unhealthy overweight and obese phenotype among the NHANES III population. Schematic diagram illustrating the statistical analysis, using periodontitis as an exposure, serum antioxidant vitamins as mediators, and MUO as an outcome.

We conducted mediation analyses to estimate the mediating role of oxidative stress in the periodontitis–MUO among the NHANES III population. As shown in Figure 2, moderate or severe periodontitis was defined as the exposure, serum antioxidant vitamins as the mediator, and metabolically unhealthy phenotype as the outcome. The mediated proportion by oxidative stress was estimated using the formula: (OR_1_−OR_2_)/(OR_1_−1) × 100, as previously reported in studies [11,33]. Given the small amount of missing data (<5%), a complete case analysis was performed, excluding participants with missing values for covariates. All analyses were conducted using R Project for Statistical Computing (version 4.2.1, Vienna, Austria), with statistical significance defined as two-sided *p* < 0.05.

## 3. Results

### 3.1. Population Characteristics

This study included 6158 participants (mean (SD) age, 43.36 (15.91); 50.9% female) in NHANES III and 2597 participants (mean (SD) age, 53.37 (14.97); 50.4% female) in KNHANES 2015. Table 1 presents the characteristics of the NHANES III population with overweight and obesity by periodontal status. Among these, 2052 (33.3%) Americans had periodontitis. Adults with periodontitis were less likely to be female and younger, with lower education and income levels, and a higher proportion of current smokers than those with periodontal health. In the NHANES III, the periodontitis group tended to diagnose diabetes, hypertension, and cardiovascular disease more than the healthy controls. Similar findings were found in KNHANES 2015 except for cardiovascular disease (Supplementary Table S1).

### 3.2. Periodontitis and MUO

Table 2 presents associations of periodontal status and MUO phenotype in the NHANES III population with overweight and obesity. Unadjusted OR for MUO phenotype was 2.037 (95% CI: 1.825 to 2.274) in participants with moderate or severe periodontitis. There was a consistent association between periodontitis and increased probability of the MUO phenotype (OR: 1.238 (1.091–1.406)) in the adjusted model. Results of all sensitivity analyses were consistent with the main analysis when applying the IDF definition of MUO (Supplementary Table S2), excluding patients with diabetes or cardiovascular disease (Supplementary Table S3), and only enrolling people with obesity (Supplementary Table S4). Further, the periodontitis–MUO association was slightly attenuated after controlling for the number of vitamins and minerals taken (OR: 1.210 (1.069–1.369), Supplementary Table S5). Similar results were obtained when current smokers were excluded from the analysis (OR: 1.226, (1.061–1.418); Supplementary Table S6). In terms of the individual components, periodontitis was more likely to be associated with hyperglycemia, elevated triglycerides, reduced HDL cholesterol, insulin resistance, and elevated systemic inflammation than in healthy controls (Table 2).

In the validation dataset (KNHANES 2015), overweight and obese participants with worse periodontal status (CPI ≥ 3 in any sextant) exhibited an increased likelihood of the MUO phenotype in the unadjusted (OR: 1.584 (1.349–1.862)) and adjusted (OR: 1.245 (1.045–1.484)) models, respectively (Supplementary Table S7). Sensitivity analyses using the IDF definition of MUO showed similar findings (Supplementary Table S8). The results were similar in participants without diabetes or cardiovascular disease (Supplementary Table S9). Among the population with obesity (BMI ≥ 27.5 kg/m^2^), however, there was no significant association between periodontitis and MUO (Supplementary Table S10). In addition, the periodontitis–MUO association was not found when the current smokers were excluded (OR: 1.152 (0.951–1.397); Supplementary Table S11). Regarding the individual components of MUO, the odds of hypertension, elevated triglycerides, reduced HDL cholesterol, and elevated systemic inflammation increased significantly in the participants with CPI ≥ 3 (Supplementary Table S7).

### 3.3. Subgroup Analyses

**Figure 3:**
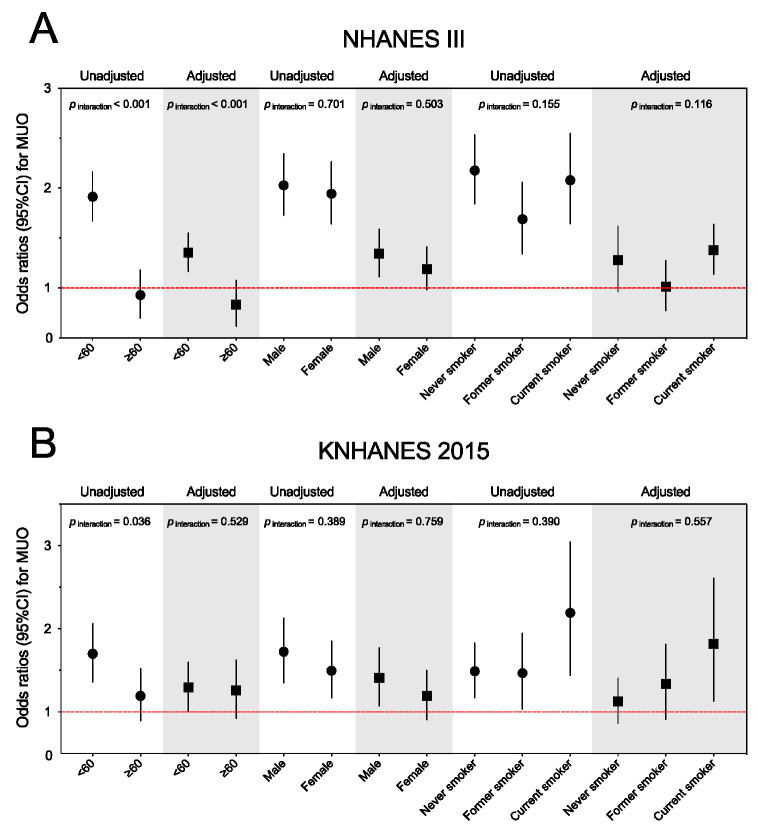
Subgroup analyses for the association between periodontitis and metabolically unhealthy overweight and obese phenotype among the NHANES III (**A**) and KNAHENS (**B**) populations by age, gender, and smoking status. Abbreviations: NHANES III, The Third National Health and Nutrition Examination Survey; KNHANES, Korean National Health And Nutrition Examination Survey; MUO, metabolically unhealthy overweight and obese; CI, confidence interval.

In the NHANES III population with overweight and obesity, the association between periodontitis and the MUO phenotype tends to be stronger in younger adults, males, or current smokers (Figure 3A). Amongst, the periodontitis by age group (<60 and ≥60 years), the interaction term was significant when added to the unadjusted and adjusted models (both *p* for interaction < 0.001). We did not find statistically significant modification effects for gender or smoking status. Similarly, the periodontitis–MUO link might be stronger in the younger adults, males, or current smokers in the KNHANES participants with overweight and obesity (Figure 3B). However, there was no statistically significant interaction in the KNHANES population (adjusted *p* for interaction = 0.529 for age group, 0.759 for gender, and 0.557 for smoking status).

### 3.4. Mediation Analyses

**Figure 4:**
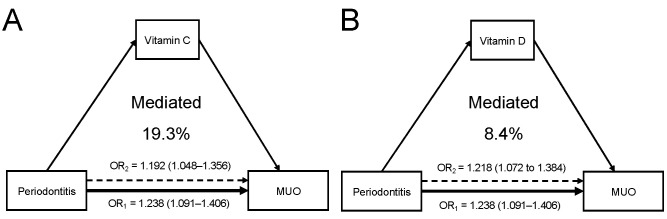
Serum antioxidant vitamins play a mediating role in the periodontitis–MUO link. (**A**) Serum vit-amin C mediated 19.3% of the association between periodontitis and MUO. (**B**) Serum vitamin D mediated 8.4% of the association.

As presented in Supplementary Table S12, moderate or severe periodontitis was reversely associated with increased levels of serum vitamin A (adjusted beta coefficient = −0.060 (−0.112 to −0.008)), vitamin C (−0.167 (−0.224 to −0.109)), vitamin D (−0.120 (−0.176 to −0.063)), and vitamin E (−0.132 (−0.179 to −0.085)). In addition, the statistically significant associations of vitamin C and D with MUO were found (Supplementary Table S13), indicating that these vitamins might play a mediating role. In the mediation analyses, the percentage mediated through serum vitamins C and D for the periodontitis–MUO association were 19.3% and 8.4%, respectively (Figure 4A,B). The sensitivity analysis that excluded current smokers revealed similar mediating effects of serum vitamins C and D (Supplementary Table S14).

## 4. Discussion

Periodontitis was positively associated with a metabolically unhealthy phenotype in the populations with overweight and obesity. Individuals with periodontitis exhibited higher dyslipidemia risk and systemic inflammation levels than those without periodontitis. We observed relatively stronger associations between periodontitis and MUO in the younger adults, males, and current smokers. The observed association persisted in the multivariable regression and was robust in several sensitivity analyses according to two nationwide population-based studies. In the mediation analyses, oxidative stress mediated the total periodontitis–MUO association, ranging between 8.4% and 19.3%.

The present study accounted for differences in body fat distribution across racial/ethnic groups and, thus, compared the relationships between periodontitis and metabolic phenotype in the US and Korean population. We focused on the metabolically unhealthy phenotype in the people with overweight and obesity. Not all obese subjects have metabolic abnormalities [5,6]. A metabolically healthy subset indicated that weight and health might not be correlated entirely. The prevalence of MetS varies in different ethnicities because of dietary and genetic differences. From 2003 to 2012, approximately 33% of the American population suffered from MetS [34]. In the Asian population, the prevalence of MetS is between 11.9% and 37.1% [35]. According to the adipose tissue overflow hypothesis, Asian populations are more susceptible to cardiometabolic complications than Western populations, even with a lower BMI [36].

Intra-abdominal fat distribution would determine the metabolic phenotype, which varies among racial/ethnic groups. In contrast to body fat mass, more attention should be paid to fat distribution in people with overweight and obesity [37]. People with high volumes of subcutaneous fat (under the body skin) are at a lower risk of cardiometabolic disease than those with excessive visceral fat (deep in the abdomen). Significant differences exist between Western and Asian populations in visceral and hepatic fat [38]. Japanese Americans have greater visceral and hepatic fat than African Americans with similar overall adiposity [38]. Our findings indicated that periodontitis increased the likelihood of MUO in the US and Korean populations, consistent with the results of previous studies in the Spanish population. In addition, we detected a significant association only among the US population with obesity (BMI ≥ 30 kg/m^2^); although the trend did not reach statistical significance in Koreans with obesity either because of a null association between periodontitis and MUO or the limited sample size. In the sensitivity analysis, the periodontitis–MUO association was not statistically significant in Koreans when excluding current smokers. A subgroup analysis also indicated that the significant association of periodontitis with MUO was found only among current smokers. We believed smoking cessation might attenuate the observed effect of periodontitis on MUO.

There is no universally accepted definition of MUO [39]. A metabolically unhealthy phenotype is usually defined as having ≥ 2 MetS components [7,13]; however, some studies adopt systemic inflammation markers and insulin resistance indices [29,30]. It was debated that patients with diabetes or heart disease were still regarded as MHO simply because they had fewer metabolic abnormalities than those defined as MUO [40]. In addition, certain definitions of MUO did not account for systemic inflammation [13]. The systemic inflammation measured by CRP might explain the disease risk related to the metabolically unhealthy phenotype [41]. Of note, Park et al. used high-sensitivity CRP to define MUO in the NHANES III study [29,30]. Periodontal inflammation has been suggested to reflect systemic inflammation and be related to cardiometabolic diseases [42,43]. Thus, numerous sensitivity analyses were conducted to minimize the bias due to considerable heterogeneity in definitions of MUO. Our findings were robust across these sensitivity analyses. When analyzing the individual components of MUO, periodontitis was associated with elevated CRP among the US and Korean populations with overweight and obesity. An interventional study in patients with obesity revealed an association between periodontal treatment and a decrease in systemic inflammation [44], consistent with the aforementioned findings. After periodontal treatment for two years, probing sites with periodontitis in patients with obesity exhibited higher levels of inflammatory biomarkers than those in patients with normal weight [45]. The increased inflammatory activity in the gingival fluid predicted the recurrence of potential periodontitis. Therefore, local and systemic inflammatory markers might affect the response to periodontal treatment in people with obesity, which needs to be confirmed by further research.

In this study, overweight and obese adults with periodontitis had lower serum vitamins C and D as well as worse metabolic profiles, suggesting the mediating role of oxidative stress. Dysregulation of the host inflammatory response to periodontal pathogens induces oxidative stress in the progression of periodontitis [46]. First, as essential organic compounds, vitamins function as antioxidants and transcription effectors for the body’s metabolic reactions. Normal vitamin D levels may impact the periodontal immune response by decreasing proinflammatory cytokine expression and the virulence of *P. gingivalis* [47,48]. In terms of vitamin C deficiency, the oral-periodontal ecosystem is altered due to an increase in oxidative stress and a susceptibility to infections [49]. In addition, the antioxidant activity of vitamin D was reported to increase the expression of superoxide dismutase and glutathione peroxidase in adipose tissue [50] and reduce the expression of NADPH oxidase and reactive oxygen species (ROS) production in glucose-treated adipocytes [51]. Second, increased ROS generation leads to the oxidative deterioration of lipids: lipid peroxidation (LPO). Patients with periodontitis had higher LPO levels in saliva and plasma compared to the healthy controls; periodontal treatment can significantly decrease salivary LPO levels [52]. LPO has been implicated in obesity-related disorders such as diabetes, atherosclerosis, hyperlipidemia, and metabolic syndrome [53,54]. Third, periodontal bacteria can disseminate to reach extraoral sites, cause bacteremia, and exacerbate metabolic alterations [55]. In turn, obesity could promote the overgrowth of bacteria associated with periodontitis. For example, *P. gingivalis*, *Fusobacterium* spp., and *T. forsythia* were in higher proportions in the patients with obesity than those without obesity [56]. Fourth, periodontitis might change food intake and dietary patterns, contributing to obesity-related metabolic disorders. Chewing capacity could be compromised in patients with periodontitis due to considerable tooth loss. Individuals with masticatory dysfunction tend to select a soft, high-fat diet, which aggravates metabolic abnormalities, rather than a fiber-rich and healthy diet [57]. In brief, a positive association between periodontitis and MUO is biologically plausible and could be partly explained by oxidative stress.

Our finding’s clinical implication lies in two aspects. First, periodontal treatment has been deemed a potential strategy for regulating systemic inflammation to prevent MetS and cardiovascular disease [58]. In a 1-year randomized controlled trial (RCT), periodontal therapy significantly reduced the CRP levels of patients with MetS [59]. Since obesity is a complex process involving genetic, behavioral, and environmental factors, care for obese adults should shift from exclusively losing weight to altering metabolic phenotypes. A systematic review indicated that overweight and obesity would not change the positive effect of periodontal treatment on inflammatory or metabolic parameters [60]. Second, antioxidant supplements could potentially prevent and treat periodontitis by inhibiting oxidative stress pathways [61]. It was reported that berries (rich in antioxidants) might improve endothelial function and promote hypoglycemia and hypolipidemic effect [62]. Given that oxidative stress is a significant risk factor shared by periodontitis and metabolic abnormalities, administering exogenous antioxidants might be a promising way of attenuating ROS-induced oxidative damage. Third, the association between periodontitis and MetS and obesity has been suggested to be causal and bidirectional [12]. In a pilot study, weight loss caused by bariatric surgery significantly improved the response to non-surgical periodontal therapy in obese patients [63]. It indicates weight loss and metabolic balance exhibited certain beneficial effects on periodontal status. In summary, overweight and obese individuals with periodontitis may benefit from periodontal treatment and exogenous antioxidants to improve their metabolic health, although the causal effect should be confirmed in future research.

Several limitations of the present study should be considered. First, the NHANES III (1988–1994) were not the latest dataset. Recently published studies also selected NHANES III to extract data since the metabolically healthy phenotype was assessed based on a fasting blood sample [9,29,64]. Although the prevalence of periodontitis, MetS, and obesity has changed over the past decades, the mechanism behind the periodontitis–MUO association is unlikely to be alerted. Second, there are different periodontal assessments and case definitions in the two national surveys. In NHANES III, we employed the CDC/AAP definition of periodontitis, whereas the CPI developed by the WHO was used to assess the periodontal status in the Korean population. A partial-mouth periodontal examination protocol would underestimate the severity of periodontitis [32]. We used the half-reduced CDC/AAP definition of periodontitis to replicate the observed association to overcome this limitation. Third, residual confounding cannot be ruled out, although we adjusted our analysis for confounding factors. Fourth, we could not confirm the causality relationship between periodontitis and MUO due to the cross-sectional study design. The clinical benefits of maintaining periodontal health on the metabolic phenotype in people with obesity needs to be further explored through clinical trials.

## 5. Conclusions

Our study demonstrated that periodontitis is associated with the metabolic unhealthy overweight and obese phenotype in the Western and Asian populations. Serum vitamins C and D, reduced by periodontitis, could be one of the biologically plausible mechanisms contributing to metabolic abnormalities in people with obesity. Further RCTs are necessary to confirm if improving periodontal health and exogenous antioxidants can be novel approaches for preventing and managing obesity-related metabolic disorders. To determine the potential bidirectional relationship between periodontitis and obesity, it is worthwhile investigating the effect of metabolic interventions (such as bariatric surgery, weight control, and dietary therapy) on periodontal health.

## Figures and Tables

**Table 1:** Characteristics of study population in NHANES III by periodontal status.

	NHANES III (1988–1994)			
Characteristic	Overall	Non/Mild Periodontitis	Moderate/Severe Periodontitis	
Number	6158	4106	2052	*p* value
Weighted number	54,909,651	39,935,703	14,973,947	
Continuous variables, mean (SD)				
Age, years	43.36 (15.91)	39.03 (14.19)	52.02 (15.66)	<0.001
BMI, kg/cm^2^	30.50 (5.08)	30.45 (5.00)	30.59 (5.20)	0.309
Waist circumference, cm	100.39 (12.00)	99.21 (11.92)	102.72 (11.77)	<0.001
SBP, mmHg	124.97 (17.64)	121.64 (15.91)	131.63 (19.01)	<0.001
DBP, mmHg	76.36 (10.46)	75.54 (10.38)	77.97 (10.43)	<0.001
Fasting glucose, mg/dL	102.68 (36.93)	98.85 (31.63)	110.21 (44.58)	<0.001
Insulin, uIU/mL ^1^	11.43 (8.61)	11.19 (8)	11.89 (9)	<0.001
HbA1c, % ^2^	5.59 (1.23)	5.43 (0.96)	5.90 (1.36)	<0.001
Total cholesterol, mg/dL	207.14 (42.66)	203.73 (42.03)	213.98 (43.07)	<0.001
HDL cholesterol, mg/dL	47.67 (13.83)	47.96 (13.67)	47.17 (14.26)	0.035
Triglycerides, mg/dL ^1^	127 (104)	121 (97)	139 (115)	<0.001
CRP, mg/L ^1^	0.21 (0.34)	0.21 (0.29)	0.21 (0.39)	<0.001
Creatinine, mg/dL ^1^	1.00 (0.30)	1.00 (0.30)	1.10 (0.30)	<0.001
Serum vitamin A, (μg/dL)	56.51 (15.99)	55.57 (15.28)	58.40 (17.17)	<0.001
Serum vitamin C, (mg/dL)	0.669 (0.42)	0.689 (0.41)	0.629 (0.424)	<0.001
Serum vitamin D, (nmol/L)	52.53 (19.74)	52.77 (19.87)	52.06 (19.48)	0.183
Serum vitamin E, (μg/dL)	1129.29 (462.66)	1107.12 (445.79)	1173.73 (491.85)	<0.001
Number of teeth ^1^	25 (6)	26 (5)	22 (9)	<0.001
Categorical, *n* (%)				
Female	3136 (50.9)	2277 (55.5)	859 (41.9)	<0.001
Race/ethnicity				
Non-Hispanic white	1890 (30.7)	1291 (31.4)	599 (29.2)	0.014
Non-Hispanic black	1824 (29.6)	1168 (28.4)	656 (32.0)	
Other	2444 (39.7)	1647 (40.1)	797 (38.8)	
Education level ^2^				
Primary school or lower	978 (16.0)	506 (12.4)	472 (23.1)	<0.001
Middle or high school graduate	3355 (54.7)	2223 (54.4)	1132 (55.5)	
College or higher	1795 (29.3)	1360 (33.3)	435 (21.3)	
Income level				
High	1535 (24.9)	506 (12.4)	472 (23.1)	<0.001
Middle	3071 (49.9)	2223 (54.4)	1132 (55.5)	
Low	1552 (25.2)	1360 (33.3)	435 (21.3)	
Smoking status ^2^				
Never smoker	3262 (53.0)	2373 (57.8)	889 (43.3)	<0.001
Former smoker	1464 (23.8)	855 (20.8)	609 (29.7)	
Current smoker	1431 (23.2)	877 (21.4)	554 (27.0)	
Diagnosed diabetes ^2^	463 (7.5)	212 (5.2)	251 (12.2)	<0.001
Diagnosed hypertension ^2^	1702 (27.9)	969 (23.8%)	733 (36.0)	<0.001
Cardiovascular disease history ^2^	286 (4.7)	118 (2.9)	168 (8.3)	<0.001
Metabolic phenotype				
Hypertension	2377 (38.6)	1295 (31.5)	1082 (52.7)	<0.001
Hyperglycemia	1913 (31.1)	1050 (25.6)	863 (42.1)	<0.001
Dyslipidemia	2392 (38.8)	1471 (35.8)	921 (44.9)	<0.001
Dyslipidemia (second criteria)	2745 (44.6)	1814 (44.2)	931 (45.4)	0.375
Insulin resistance	615 (10.0)	327 (8.0)	288 (14.0)	<0.001
Systemic inflammation	653 (10.6)	407 (9.9)	246 (12.0)	<0.001
Metabolically healthy status				
MHO	2959 (48.1)	2213 (53.9)	746 (36.4)	<0.001
MUO	3199 (51.9)	1893 (46.1)	1306 (63.6)	

^1^ Non-normal distribution continuous variable, median (interquartile range). Independent-Samples Mann–Whitney U Test was used to compare the non-parametric variables between two groups. ^2^ Missing values in NHANES III (*n* = 6158): HbA1C (*n* = 29; 0.5%), education level (*n* = 30; 0.5%), smoking status (*n* = 1; 0.016%), diagnosed diabetes (*n* = 7; 0.1%), diagnosed hypertension (*n* = 47; 0.8%), cardiovascular disease history (*n* = 69; 1.1%). All *p*-values were calculated with a two-sided significance level of 0.05. Abbreviations: NHANES III, The Third National Health and Nutrition Examination Survey; SD, standard deviation; BMI, body mass index; SBP, systolic blood pressure; DBP, diastolic blood pressure; HbA1c, glycohemoglobin A1c; HDL, high-density lipoprotein; CRP, C-reactive protein; MHO, metabolically healthy overweight and obese; MUO, metabolically unhealthy overweight and obese.

**Table 2:** Weighted associations of periodontitis with metabolically unhealthy overweight and obese phenotype and the individual components in the NHANES III population.

Variables	Case/Participants	Unadjusted OR (95% CI)	Adjusted OR (95% CI) ^1^
Metabolically unhealthy overweight/obese (MUO)			
Non/mild periodontitis	1893/4106	1	1
Moderate/severe periodontitis	1306/2052	2.037 (1.825 to 2.274)	1.238 (1.091 to 1.406)
Hypertension			
Non/mild periodontitis	1295/4106	1	1
Moderate/severe periodontitis	1082/2052	2.409 (2.158 to 2.688)	1.084 (0.948 to 1.240)
Hyperglycemia			
Non/mild periodontitis	1050/4106	1	1
Moderate/severe periodontitis	863/2052	2.114 (1.888 to 2.368)	1.168 (1.024 to 1.333)
Elevated triglycerides			
Non/mild periodontitis	1471/4106	1	1
Moderate/severe periodontitis	921/2052	1.452 (1.302 to 1.619)	1.168 (1.022 to 1.334)
Reduced HDL cholesterol			
Non/mild periodontitis	1814/4106	1	1
Moderate/severe periodontitis	931/2052	1.058 (0.950 to 1.178)	1.163 (1.028 to 1.316)
HOMA-IR (≥ 90th percentile)			
Non/mild periodontitis	327/4106	1	1
Moderate/severe periodontitis	288/2052	1.881 (1.588 to 2.227)	1.460 (1.205 to 1.770)
CRP (≥ 90th percentile)			
Non/mild periodontitis	407/4106	1	1
Moderate/severe periodontitis	246/2052	1.242 (1.049 to 1.470)	1.372 (1.129 to 1.666)

^1^ Model was adjusted for age, gender, race/ethnicity, total cholesterol, creatinine, number of teeth, education level, income level, smoking status, and cardiovascular disease history. Abbreviations: NHANES III, The Third National Health and Nutrition Examination Survey; HDL, high-density lipoprotein; HOMA-IR, homoeostasis model assessment of insulin resistance; CRP, C-reactive protein; OR, odds ratio; CI, confidence interval.

## Data Availability

The datasets generated and analyzed during the current study are publicly available in the repository of NHANES (https://wwwn.cdc.gov/nchs/nhanes/, accessed on 14 September 2022) and KNHANES (https://kdca.go.kr/, accessed on 14 September 2022).

## Supplementary Materials

The following supporting information can be downloaded at: https://www.mdpi.com/article/10.3390/nu14224939/s1, Table S1. Characteristics of study population in KNHANES 2015 by periodontal status; Table S2. Sensitivity analysis for association between periodontitis and the metabolically unhealthy phenotype among populations with overweight and obesity in the NHANES III; Table S3. Sensitivity analysis for association between periodontitis and the metabolically unhealthy phenotype among populations with overweight and obesity in the NHANES III; Table S4. Sensitivity analysis for association between periodontitis and the metabolically unhealthy phenotype among populations with obesity in the NHANES III; Table S5. Sensitivity analysis for association between periodontitis and the metabolically unhealthy phenotype among populations with overweight and obesity in the NHANES III; Table S6. Sensitivity analysis for association between periodontitis and the metabolically unhealthy phenotype among population with overweight and obesity in the NHANES III; Table S7. Weighted associations of periodontitis with the metabolically unhealthy overweight and obese phenotype and the individual components in the KNHANES 2015; Table S8. Sensitivity analysis for association between periodontitis and the metabolically unhealthy phenotype among populations with overweight and obesity in the KNHANES 2015; Table S9. Sensitivity analysis for association between periodontitis and the metabolically unhealthy phenotype among populations with overweight and obesity in the KNHANES 2015; Table S10. Sensitivity analysis for association between periodontitis and the metabolically unhealthy phenotype among populations with obesity in the KNHANES 2015; Table S11. Sensitivity analysis for association between periodontitis and the metabolically unhealthy phenotype among populations with overweight and obesity in the KNHANES 2015; Table S12. Association between moderate and severe periodontitis and serum antioxidant vitamins in the NHANES III; Table S13. Association between serum antioxidant vitamins and MUO in the NHANES III; Table S14. Sensitivity analysis for the mediating effect of serum vitamins on the periodontitis–MUO association in the NHANES III.
