# Supplementary material for: Serum Antioxidant Vitamins Mediate the Association between Periodontitis and Metabolically Unhealthy Overweight/Obesity
Source: Nutrients. 2022 Nov 21;14(22):4939. doi: 10.3390/nu14224939 (PMC9694708; doi:10.3390/nu14224939)
Supplement: Supplementary file 1 [file nutrients-14-04939-s001.zip › nutrients-2043022-supplementary.pdf]

nutrients

MDPI

Article

Serum Antioxidant VitaminsMediate the Association between Periodontitis and Metabolically Unhealthy Overweight/Obesity

An Li [1,2,+], Zhi Tang [3,+], Peijun Zhu [1], Florien van den Bosch [2], Yuntao Chen [4,5], Shulan Xu [1,*] and Geerten-Has E. Tjakkes [2]

TABLE OF CONTENTS

Table S1. Characteristics of study population in KNHANES 2015 by periodontal status3

Table S2. Sensitivity analysis for association between periodontitis and metabolically unhealthy phenotype among population with overweight/obesity in the NHANES III [a] 4

Table S3. Sensitivity analysis for association between periodontitis and metabolically unhealthy phenotype among population with overweight/obesity in the NHANES III [a] 4

Table S4. Sensitivity analysis for association between periodontitis and metabolically unhealthy phenotype among population with obesity in the NHANES III [a] 4

Table S5. Sensitivity analysis for association between periodontitis and metabolically unhealthy phenotype among population with overweight/obesity in the NHANES III................4

Table S6. Sensitivity analysis for association between periodontitis and metabolically unhealthy phenotype among population with overweight/obesity in the NHANES III [a] 5

Table S7. Weighted associations of periodontitis with metabolically unhealthy overweight/obese phenotype and the individual components in the KNHANES 2015 5

Table S8. Sensitivity analysis for association between periodontitis and metabolically unhealthy phenotype among population with overweight/obesity in the KNHANES 2015 [a].......5

Table S9. Sensitivity analysis for association between periodontitis and metabolically unhealthy phenotype among population with overweight/obesity in the KNHANES 2015 [a].......6

Table S10. Sensitivity analysis for association between periodontitis and metabolically unhealthy phenotype among population with obesity in the KNHANES 2015 [a].............................6

Table S11. Sensitivity analysis for association between periodontitis and metabolically unhealthy phenotype among population with overweight/obesity in the KNHANES 2015 [a].......6

Table S12. Association between moderate/severe periodontitis and serum antioxidant vitamins in the NHANES III [a]..6

Table S13. Association between serum antioxidant vitamins and MUO in the NHANES III [a] 7

Table S14. Sensitivity analysis for the mediating effect of serum vitamins on the periodontitis–MUO association in the NHANES III [a] 7

Supplementary Table S1. Characteristics of study population in KNHANES 2015 by periodontal status.

	KNHANES 2015			
	**Overall**	**CPI 0–2**	**CPI 3–4**	*p* Value
Number	2597	1580	1017	
Weighted number	15312417	9182536	6129881	
Continuous variables, mean (SD)				
Age, years	53.37 (14.97)	50.50 (15.77)	57.85 (12.39)	< 0.001
BMI, kg/cm²	26.20 (2.68)	26.13 (2.69)	26.31 (2.67)	0.095
Waist circumference, cm [b]	88.56 (7.83)	88.03 (7.92)	89.37 (7.61)	< 0.001
SBP, mmHg	122.40 (16.61)	120.29 (15.72)	125.71 (17.43)	< 0.001
DBP, mmHg	77.18 (10.24)	76.79 (9.98)	77.80 (10.60)	0.013
Fasting glucose, mg/dL	104.90 (27.60)	102.00 (21.69)	109.41 (34.37)	< 0.001
Insulin, uIU/mL [a]	8.10 (6.20)	8.10 (6.20)	8.00 (6.20)	0.502
HbA1c, % [b]	5.80 (0.80)	5.71 (0.687)	5.94 (0.929)	< 0.001
Total cholesterol, mg/dL	193.34 (36.59)	193.57 (35.579)	193.00 (38.13)	0.701
HDL cholesterol, mg/dL	47.85 (11.76)	48.69 (11.837)	46.54 (11.523)	< 0.001
Triglycerides, mg/dL [a]	128 (95)	123.5 (91)	135 (100.5)	< 0.001
CRP, mg/L [a]	0.70 (1.00)	0.68 (0.90)	0.72 (1.05)	0.005
Creatinine, mg/dL [a]	0.84 (0.28)	0.83 (0.27)	0.85 (0.28)	0.057
Number of teeth [a]	28 (1.00)	28 (1.0)	28 (1.00)	< 0.001
Categorical, *n* (%)				
Gender (% female)	1310 (50.4)	839 (53.1)	471 (46.3)	0.001
Education level				
Primary school or lower	610 (23.5)	324 (20.5)	286 (28.1)	< 0.001
Middle/high school graduate	1241 (47.8)	726 (45.9)	515 (50.6)	
College or higher	746 (28.7)	530 (33.5)	216 (21.2)	
Income level [b]				
High	632 (24.4)	406 (25.8)	226 (22.4)	0.014
Middle	1303 (50.4)	802 (50.9)	501 (49.6)	
Low	650 (25.1)	367 (23.3)	283 (28.0)	
Smoking status [b]				
Never smoker	1408 (55.2)	915 (59.0）	493 (49.4)	< 0.001
Former smoker	651 (25.5)	371 (23.9)	280 (28.1)	
Current smoker	490 (19.2)	265 (17.1)	225 (22.5)	
Diagnosed diabetes [b]	260 (10.5)	136 (9.0)	124 (12.9)	0.002
Diagnosed hypertension	824 (31.7)	418 (26.5)	406 (39.9)	< 0.001
Cardiovascular disease history	263 (10.1)	163 (10.3)	100 (9.8)	0.690
Metabolic phenotype				
Hypertension	901 (34.7)	485 (30.7)	416 (40.9)	< 0.001
Hyperglycemia	1166 (44.9)	650 (41.1)	516 (50.7)	< 0.001
Dyslipidemia	982 (37.8)	557 (35.3)	425 (41.8)	0.001
Dyslipidemia (second criteria)	1113 (42.9)	645 (40.8)	468 (46.0)	0.009
Insulin resistance	259 (10.0)	146 (9.2)	113 (11.1)	0.120
Systemic inflammation	259 (10.0)	130 (8.2)	129 (12.7)	< 0.001
Metabolically healthy status				
MHO	1153 (44.4)	771 (48.8)	382 (37.6)	< 0.001
MUO	1444 (55.6)	809 (51.2)	635 (62.4)	

[a] Non-normal distribution continuous variable, median (interquartile range). Independent-Samples Mann-Whitney U Test was used to compare the non-parametric variables between two groups. [b] Missing values in KNHANES 2015 (*n* = 2597): waist circumference (*n* = 1; 0.039%), HbA1C (*n* = 12;

0.5%), income level (n = 12; 0.5%), smoking status (n = 48; 1.8%), diagnosed diabetes (n = 121; 4.7%). All p-values were calculated with a two-sided significance level of 0.05. **Abbreviations**: KNHANES, Korean National Health And Nutrition Examination Survey; CPI, community periodontal index; SD, standard deviation; BMI, body mass index; SBP, systolic blood pressure; DBP, diastolic blood pressure; HbA1c, glycohaemoglobin A1c; HDL, high-density lipoprotein; CRP, C-reactive protein; MHO, metabolically healthy overweight/obese; MUO, metabolically unhealthy overweight/obese.

Supplementary Table S2. Sensitivity analysis for association between periodontitis and metabolically unhealthy phenotype among population with overweight/obesity in the NHANES III [a].

	Case/ participants	Unadjusted OR (95% CI)	Adjusted OR (95% CI) [b]
Non/mild periodontitis	1721/4106	1 [Reference]	1 [Reference]
Moderate/severe periodontitis	1215/2052	**2.006 (1.800 to 2.236)**	**1.179 (1.038 to 1.339)**

[a] Metabolically unhealthy phenotype was defined by IDF consensus definition. [b] Adjusted model was adjusted for age, gender, race/ethnicity, total cholesterol, creatinine, number of teeth, education level, income level, smoking status, and cardiovascular disease history. Bold indicates p value < 0.05. **Abbreviations**: NHANES III, The Third National Health and Nutrition Examination Survey; IDF, International Diabetes Federation; OR, odds ratio; CI, confidence interval.

Supplementary Table S3. Sensitivity analysis for association between periodontitis and metabolically unhealthy phenotype among population with overweight/obesity in the NHANES III [a].

	Case/ participants	Unadjusted OR (95% CI)	Adjusted OR (95% CI) [b]
Non/mild periodontitis	1643/3750	1 [Reference]	1 [Reference]
Moderate/severe periodontitis	980/1651	**1.870 (1.662 to 2.104)**	**1.201 (1.050 to 1.375)**

[a] We excluded individuals with diabetes or cardiovascular disease. [b] Adjusted model was adjusted for age, gender, race/ethnicity, total cholesterol, creatinine, number of teeth, education level, income level, and smoking status. Bold indicates p value < 0.05. **Abbreviations**: NHANES III, The Third National Health and Nutrition Examination Survey; OR, odds ratio; CI, confidence interval.

Supplementary Table S4. Sensitivity analysis for association between periodontitis and metabolically unhealthy phenotype among population with obesity in the NHANES III [a].

	Case/ participants	Unadjusted OR (95% CI)	Adjusted OR (95% CI) [b]
Non/mild periodontitis	986/1742	1 [Reference]	1 [Reference]
Moderate/severe periodontitis	642/890	**1.972 (1.654 to 2.352)**	**1.356 (1.113 to 1.653)**

[a] Population with obesity was defined by body-mass index ≥30 kg/m^2. [b] Adjusted model was adjusted for age, gender, race/ethnicity, total cholesterol, creatinine, number of teeth, education level, income level, smoking status, and cardiovascular disease history. Bold indicates p value < 0.05. **Abbreviations**: NHANES III, The Third National Health and Nutrition Examination Survey; OR, odds ratio; CI, confidence interval.

Supplementary Table S5. Sensitivity analysis for association between periodontitis and metabolically unhealthy phenotype among population with overweight/obesity in the NHANES III.

	Case/ participants	Unadjusted OR (95% CI)	Adjusted OR (95% CI) [a]
Non/mild periodontitis	1893/4106	1 [Reference]	1 [Reference]
Moderate/severe periodontitis	1306/2052	**2.037 (1.825 to 2.274)**	**1.210 (1.069 to 1.369)**

[a] Adjusted model was adjusted for age, gender, race/ethnicity, total cholesterol, creatinine, number of teeth, education level, income level, smoking status, cardiovascular disease history, and the number of vitamins/minerals taken. Bold indicates p value < 0.05. **Abbreviations**: NHANES III, The Third National Health and Nutrition Examination Survey; OR, odds ratio; CI, confidence interval.

Supplementary Table S6. Sensitivity analysis for association between periodontitis and metabolically unhealthy phenotype among population with overweight/obesity in the NHANES III [a].

	Case/ participants	Unadjusted OR (95% CI)	Adjusted OR (95% CI) [b]
Non/mild periodontitis	1492/3228	1 [Reference]	1 [Reference]
Moderate/severe periodontitis	955/1498	**2.037 (1.794 to 2.313)**	**1.226 (1.061 to 1.418)**

[a] Current smokers were excluded. [b] Adjusted model was adjusted for age, gender, race/ethnicity, total cholesterol, creatinine, number of teeth, education level, income level, and cardiovascular disease history. Bold indicates p value < 0.05. **Abbreviations**: NHANES III, The Third National Health and Nutrition Examination Survey; OR, odds ratio; CI, confidence interval.

Supplementary Table S7. Weighted associations of periodontitis with metabolically unhealthy overweight/obese phenotype and the individual components in the KNHANES 2015.

	Case/ Participants	Unadjusted OR (95% CI)	Adjusted OR (95% CI) [a]
Metabolically unhealthy overweight/obese (MUO)			
CPI 0–2	809/1580	1 [Reference]	1 [Reference]
CPI 3–4	635/1017	**1.584 (1.349 to 1.862)**	**1.245 (1.045 to 1.484)**
Hypertension			
CPI 0–2	485/1580	1 [Reference]	1 [Reference]
CPI 3–4	416/1017	**1.559 (1.322 to 1.839)**	**1.196 (1.002 to 1.428)**
Hyperglycemia			
CPI 0–2	650/1580	1 [Reference]	1 [Reference]
CPI 3–4	516/1017	**1.482 (1.265 to 1.738)**	1.134 (0.949 to 1.354)
Elevated triglycerides			
CPI 0–2	557/1580	1 [Reference]	1 [Reference]
CPI 3–4	425/1017	**1.324 (1.126 to 1.558)**	**1.191 (1.001 to 1.417)**
Reduced HDL cholesterol			
CPI 0–2	645/1580	1 [Reference]	1 [Reference]
CPI 3–4	468/1017	**1.242 (1.059 to 1.456)**	**1.220 (1.026 to 1.449)**
HOMA-IR (≥90th percentile)			
CPI 0–2	146/1580	1 [Reference]	1 [Reference]
CPI 3–4	113/1017	1.242 (0.958 to 1.611)	1.191 (0.900 to 1.576)
CRP (≥90th percentile)			
CPI 0–2	130/1580	1 [Reference]	1 [Reference]
CPI 3–4	129/1017	**1.645 (1.272 to 2.128)**	**1.367 (1.044 to 1.788)**

[a] Adjusted model was adjusted for age, gender, total cholesterol, creatinine, number of teeth, education level, income level, smoking status, and cardiovascular disease history. Bold indicates p value < 0.05. **Abbreviations**: KNHANES, Korean National Health And Nutrition Examination Survey; CPI, community periodontal index; HDL, high-density lipoprotein; HOMA-IR, homoeostasis model assessment of insulin resistance; CRP, C-reactive protein. OR, odds ratio; CI, confidence interval.

Supplementary Table S8. Sensitivity analysis for association between periodontitis and metabolically unhealthy phenotype among population with overweight/obesity in the KNHANES 2015 [a].

	Case/ participants	Unadjusted OR (95% CI)	Adjusted OR (95% CI)

CPI 0-2	735/1580	1 [Reference]	1 [Reference]
CPI 3-4	595/1017	**1.620 (1.381 to 1.901)**	**1.278 (1.078 to 1.516)**

[a] Metabolically unhealthy phenotype was defined by IDF consensus definition. [b] Adjusted Model was adjusted for age, gender, total cholesterol, creatinine, number of teeth, education level, income level, smoking status, and cardiovascular disease history. Bold indicates p value < 0.05. **Abbreviations**: KNHANES, Korean National Health And Nutrition Examination Survey; IDF, International Diabetes Federation; CPI, community periodontal index; OR, odds ratio; CI, confidence interval.

Supplementary Table S9. Sensitivity analysis for association between periodontitis and metabolically unhealthy phenotype among population with overweight/obesity in the KNHANES 2015 [a].

	**Case/ participants**	**Unadjusted OR (95% CI)**	**Adjusted OR (95% CI)**
CPI 0-2	588/1236	1 [Reference]	1 [Reference]
CPI 3-4	444/754	**1.632 (1.376 to 1.934)**	**1.269 (1.058 to 1.522)**

[a] We excluded individuals with diabetes or cardiovascular disease. [b] Adjusted model was adjusted for age, gender, total cholesterol, creatinine, number of teeth, education level, income level, and smoking status. Bold indicates p value < 0.05. **Abbreviations**: KNHANES, Korean National Health And Nutrition Examination Survey; CPI, community periodontal index; OR, odds ratio; CI, confidence interval.

Supplementary Table S10. Sensitivity analysis for association between periodontitis and metabolically unhealthy phenotype among population with obesity in the KNHANES 2015 [a].

	**Case/ participants**	**Unadjusted OR (95% CI)**	**Adjusted OR (95% CI) [b]**
CPI 0-2	262/376	1 [Reference]	1 [Reference]
CPI 3-4	185/253	1.184 (0.831 to 1.687)	1.123 (0.806 to 1.565)

[a] Population with obesity was defined by body-mass index ≥27.5 kg/m^2. [b] Adjusted Model was adjusted for age, gender, total cholesterol, creatinine, number of teeth, education level, income level, smoking status, and cardiovascular disease history. Bold indicates p value < 0.05. **Abbreviations**: KNHANES, Korean National Health And Nutrition Examination Survey; CPI, community periodontal index; OR, odds ratio; CI, confidence interval.

Supplementary Table S11. Sensitivity analysis for association between periodontitis and metabolically unhealthy phenotype among population with overweight/obesity in the KNHANES 2015 [a].

	**Case/ participants**	**Unadjusted OR (95% CI)**	**Adjusted OR (95% CI) [b]**
CPI 0-2	652/1286	1 [Reference]	1 [Reference]
CPI 3-4	465/773	**1.468 (1.225 to 1.759)**	1.152 (0.951 to 1.397)

[a] Current smokers were excluded. [b] Adjusted model was adjusted for age, gender, total cholesterol, creatinine, number of teeth, education level, income level, and cardiovascular disease history. Bold indicates p value < 0.05. **Abbreviations**: KNHANES, Korean National Health And Nutrition Examination Survey; CPI, community periodontal index; OR, odds ratio; CI, confidence interval.

Supplementary Table S12. Association between moderate/severe periodontitis and serum antioxidant vitamins in the NHANES III [a].

**Moderate/severe periodontitis**	**Unadjusted beta coefficient (95% CI)**	**Adjusted beta coefficient (95% CI) [b]**
Serum vitamin A	**0.174 (0.121 to 0.228)**	**-0.060 (-0.112 to -0.008)**

Serum vitamin C	**-0.152 (-0.206 to -0.098)**	**-0.167 (-0.224 to -0.109)**
Serum vitamin D	-0.038 (-0.091 to 0.016)	**-0.120 (-0.176 to -0.063)**
Serum vitamin E	**0.138 (0.085 to 0.192)**	**-0.132 (-0.179 to -0.085)**

[a] The serum antioxidant vitamins were transformed as Z-score and regarded as the outcome variable in the linear regression models. [b] Adjusted Model was adjusted for age, gender, total cholesterol, creatinine, number of teeth, education level, income level, smoking status, and cardiovascular disease history. Bold indicates p value < 0.05. **Abbreviations**: NHANES III, The Third National Health and Nutrition Examination Survey; CI, confidence interval.

Supplementary Table S13. Association between serum antioxidant vitamins and MUO in the NHANES III [a].

MUO	Unadjusted OR (95% CI)	Adjusted OR (95% CI)
Serum vitamin A	**1.351 (1.281 to 1.425)**	0.899 (0.777 to 1.041)
Serum vitamin C	**0.882 (0.837 to 0.928)**	**0.827 (0.780 to 0.877)**
Serum vitamin D	0.963 (0.916 to 1.013)	**0.830 (0.780 to 0.883)**
Serum vitamin E	**1.912 (1.781 to 2.053)**	0.966 (0.833 to 1.121)

[a] The serum antioxidant vitamins were transformed as Z-score and regarded as the exposure variable in the Logistic regression models. [b] Adjusted Model was adjusted for age, gender, total cholesterol, creatinine, number of teeth, education level, income level, smoking status, and cardiovascular disease history. Bold indicates p value < 0.05. **Abbreviations**: NHANES III, The Third National Health and Nutrition Examination Survey; MUO, metabolically unhealthy overweight/obese; OR, odds ratio; CI, confidence interval.

Supplementary Table S14. Sensitivity analysis for the mediating effect of serum vitamins on the periodontitis–MUO association in the NHANES III [a].

	Cases/ Participants	Model 1 [a] OR1 (95% CI)	Model 2 [a] OR2 (95% CI)	% excess risk explained [b]
Vitamin C				
Non/mild periodontitis	1492/3228	1 [Reference]	1 [Reference]	22.1%
Moderate/severe periodontitis	955/1498	1.226 (1.061 to 1.418) **	1.176 (1.014 to 1.363 *	
Vitamin D				
Non/mild periodontitis	1492/3228	1 [Reference]	1 [Reference]	8.8%
Moderate/severe periodontitis	955/1498	1.226 (1.061 to 1.418) **	1.206 (1.042 to 1.396) *	

[a] Current smokers were excluded. [b] Model 1 was adjusted for age, gender, race/ethnicity, total cholesterol, creatinine, number of teeth, education level, income level, and cardiovascular disease history. Model 2 was further adjusted for serum vitamins. The formula $\frac{(OR1-OR2)}{(OR1-1)} \times 100$ was used to determine the percentage of excess risk explained by serum vitamins, where OR1 is the hazard ratio of Model 1 and OR2 is the hazard ratio of Model 2. * indicates p <0.05; ** indicates p <0.01; *** indicates p <0.001. **Abbreviation**: NHANES III, The Third National Health and Nutrition Examination Survey; OR, odds ratio; CI, confidence interval.
